# Using a multistep approach with multidisciplinary team to increase the diagnosis rate of Lynch syndrome-associated colorectal cancer after universal screening: a single-center study in Japan

**DOI:** 10.1186/s13053-023-00258-0

**Published:** 2023-07-17

**Authors:** Kyota Tatsuta, Mayu Sakata, Moriya Iwaizumi, Risa Kojima, Katsumasa Yamanaka, Satoshi Baba, Katsunori Suzuki, Yoshifumi Morita, Hirotoshi Kikuchi, Yoshihiro Hiramatsu, Kiyotaka Kurachi, Hiroya Takeuchi

**Affiliations:** 1 Department of Surgery, Hamamatsu University School of Medicine, 1-20-1, Handayama, Higashi-ku, Hamamatsu, 431-3192 Shizuoka Japan; 2 Department of Laboratory Medicine, Hamamatsu University School of Medicine, 1-20-1, Handayama, Higashi-ku, Hamamatsu, 431-3192 Shizuoka Japan; 3 Clinical & Molecular Genetics Center, Hamamatsu University School of Medicine, 1-20-1, Handayama, Higashi-ku, Hamamatsu, 431-3192 Shizuoka Japan; 4 Department of Diagnostic Pathology, Hamamatsu University School of Medicine, 1-20-1, Handayama, Higashi-ku, Hamamatsu, 431-3192 Shizuoka Japan; 5 Department of Perioperative Functioning Care and Support, Hamamatsu University School of Medicine, 1-20-1, Handayama, Higashi-ku, Hamamatsu, 431-3192 Shizuoka Japan

**Keywords:** Lynch syndrome, Universal screening, Genetic counseling, Multidisciplinary team management

## Abstract

**Backgrounds:**

: This study aimed to evaluate the changes in the rates of genetic counseling and genetic testing as well as the diagnosis rate of Lynch syndrome (LS)-associated colorectal cancer before and after multistep approach with multidisciplinary team in Japanese.

**Methods:**

In September 2016, we started universal screening for LS by mismatch repair protein immunohistochemistry and prospectively collected the records. Following patient interviews, we started multistep approach with multidisciplinary team (MA) in January 2020. MA consists of six surgeons, one genetic counselor, one medical geneticist, and six pathologists. MA is set up to compensate for patients’ lack of knowledge about genetic diseases and make case selection for elderly colorectal cancer patients with deficient mismatch repair (dMMR). MA is designed as a system that could be performed by a small number of medical genetic specialists. A total of 522 patients were included during the study duration, 323 and 199 patients in the pre-MA (P-MA) and MA groups, respectively.

**Results:**

The frequency of dMMR in all patients was 10.0%. The patient interview results indicated a significant lack of patient education regarding genetic diseases. The rates of genetic counseling and genetic testing was significantly higher in MA group than in P-MA group (genetic counseling: MA 34.6% vs. P-MA 7.7%, p = 0.04; genetic testing: MA 30.8% vs. P-MA 3.8%, p = 0.02). Moreover, the diagnosis rate of LS-associated colorectal cancer was significantly higher in MA group (2.5%) than in P-MA group (0.3%) (*P* = 0.03). In addition, MA could be performed without problems despite the small number of medical and human genetics specialists.

**Conclusions:**

MA has achieved appropriate pickup of suspected hereditary colorectal cancer patients and complemented the lack of knowledge about genetic diseases. The introduction of MA increased LS-associated colorectal cancer after universal screening. MA is an appropriate LS screening protocol for Japanese patients who lag behind in medical and human genetics education.

**Supplementary Information:**

The online version contains supplementary material available at 10.1186/s13053-023-00258-0.

## Background

Lynch syndrome (LS) is the most common cause of hereditary colorectal cancer (CRC), inherited by autosomal dominant mutations in mismatch repair (MMR) genes, *MLH1*, *MSH2*, *MSH6*, and *PMS2*, or *EpCAM* gene [1–4]. Universal screening for LS has shown to be cost-effective owing to its ability to prevent secondary cancers in affected individuals as well as primary cancers in at-risk relatives [5, 6]. The latest guidelines recommended universal screening for LS in patients with CRC and endometrial cancer [7, 8].

Its feasibility of universal screening for LS has been well demonstrated in research and clinical settings. LS screening protocols have been more likely introduced in research and academic centers, and less likely introduced in community hospitals [9, 10]. However, in Japan, where universal screening for LS is not common, LS screening protocols are not well developed even at academic centers. One of the reasons is that medical and human genetics education was inadequate until recently, resulting in lack of patient knowledge about genetic diseases and very few medical and human genetics specialists at several Japanese medical institutes [11].

Currently, in Japan, an LS screening protocol was developed based on Western studies [12, 13]. Although this protocol has a strong scientific basis, it requires multiple expensive tests, such as *BRAF* V600E variant or *MLH1* promoter methylation. In Japan, the choice of whether to undergo these tests is up to the patients [12, 13]. Many Japanese patients do not fully understand the importance of these tests because they have less access to sufficient explanations about genetic diseases. As a result, many Japanese patients do not wish to performe these tests. Therefor, we hypothesized that a new LS screening protocol suitable for Japanese was needed.

We developed a multistep approach with a small number of multidisciplinary team for the purpose of creating an LS screening protocol suitable for Japanese. This study aimed to evaluate the changes in the rates of genetic counseling and genetic testing as well as the diagnostic rate of LS-associated CRC before and after the multistep approach with multidisciplinary team.

## Methods

### Study design and patient population

The study design was approved by the institutional review board of Hamamatsu University School of Medicine (IRB number:16–138). We prospectively collected the records of 566 patients who underwent colorectal surgery between September 2016 and November 2021 at Hamamatsu University School of Medicine. Among them, 24 patients who underwent endoscopic resection at the other hospitals and 20 patients who did not give consent to participate in this study were excluded. Finally, 522 patients were included.

### Immunohistochemistry for MMR proteins

In September 2016, we started to perform universal screening using immunohistochemistry (IHC) for four MMR proteins (MLH1, MSH2, MSH6, and PMS2) according to the manufacturer’s protocol, in 4-µm-thick formalin-fixed paraffin-embedded sections. MMR proteins (MMRP) was evaluated using endoscopically resected specimens or surgically resected specimens.

The primary antibodies used for detecting the MMRP were anti-hMLH1 antibody (clone G168-728; BD Biosciences; 1:100), anti-hMSH2 antibody (clone FE11; Calbiochem; 1:100), anti-MSH6 antibody (clone 44/MSH6; BD Biosciences; 1:100), and anti-hPMS2 antibody (clone A16-4; BD Biosciences; 1: 100) until June 2021. From July 2021, fully automated IHC and in situ hybridization slide-staining system began to be used. Accordingly, the VENTANA detection kits were used for evaluation, anti-MLH1 antibody (clone M1; Roche), anti-MSH2 antibody (clone G219-1129; Roche), anti-MSH6 antibody (clone SP93; Roche), and anti-PMS2 antibody (clone A16-4; Roche).

The normal staining patterns for MLH1, MSH2, MSH6, and PMS2 are unclear. However, the absence of nuclear staining in the tumor cells in the presence of nuclear staining of non-neoplastic cells, such as normal colonic epithelial cells, lymphocytes, or stromal cells, was considered to represent an abnormal pattern. This evaluation method is performed in accordance with the Japanese Society for Cancer of the Colon and Rectum Guidelines 2016 and 2020 for the Clinical Practice of Hereditary Colorectal Cancer [12, 13]. The staining results were evaluated by consensus between two independent pathologists. Tumors with loss of expression of either protein were designated as deficient MMR (dMMR).

### Pre-multistep approach with multidisciplinary team (P-MA)

**Fig. 1:**
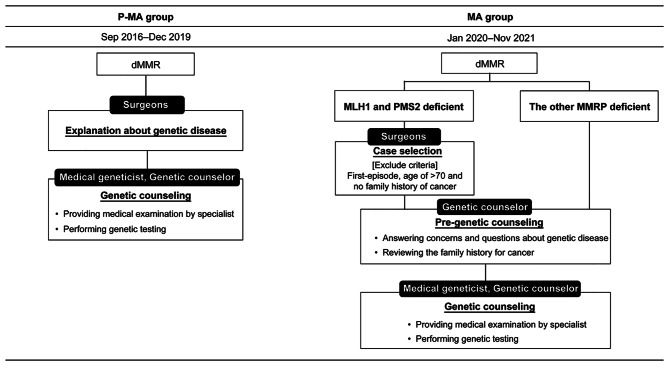
Flow charts of the universal screening for Lynch syndrome over time In the pre-multidisciplinary team management group, only surgeons explained the possibility of LS and referred patients to receive genetic counseling. In the multidisciplinary team management group, surgeons, genetic counselors, and medical geneticists each have their own role dMMR: deficient MMR, LS: Lynch syndrome, CRC: colorectal cancer

Before a multistep screening with multidisciplinary team, the surgeons explained the possibility of LS to all patients diagnosed with dMMR. Only those who make a request received genetic counseling by medical geneticists (Fig. 1). On the other hand, we interviewed all patients who did not receive genetic counseling to know why they refused it.

### Multistep approach with multidisciplinary team (MA)

Based on the result of the interviews, we started a multidisciplinary team in January 2020. A multidisciplinary team consists of six surgeons, one genetic counselor, one medical geneticist, and six pathologists.

Figure 1 showed the comparison of a medical practice flow chart for dMMR patients before and after MA. As the first step, the pathologist and surgeon confirmed the pattern of protein expression loss. When a tumor showed loss of MLH1 and PMS2 expression, the surgeons determined whether the following exclusion criteria were applied or not. Exclusion criteria were first-episode CRC, 70 years old and above at CRC diagnosis, and no family history of cancer. For the remaining cases, the surgeons refer the patient for hereditary CRC and recommend that the patients undergo pre-genetic counseling with a genetic counselor on the same day or within a week. In the case of a protein expression loss pattern other than MLH1 and PMS2, the surgeons refer the patient for hereditary CRC and recommend that the patients undergo pre-genetic counseling with a genetic counselor as well. As the second step, the genetic counselor performed free-of-charge pre-genetic counseling by reviewing the family history for cancer, outlining the genetic disease, listening to the patient’s concerns, and explaining the flow of medical examination. Then, genetic counseling was offered to patients who requested specialist medical examination following pre-genetic counseling. After genetic counseling by medical geneticists and genetic counselor, patients underwent genetic testing, which was performed on peripheral blood samples.

### Assessment of MA performance

In this study, patients were divided into the following two groups: P-MA group for patients before multistep approach with multidisciplinary team and MA group for those after multistep approach with multidisciplinary team. We evaluated the changes in the rates of genetic counseling and genetic testing as well as the diagnostic rate of LS-associated CRC between P-MA and MA groups.

### Statistical analysis

Statistical analyses were conducted using JMP® 16 (SAS Institute Inc., Cary, NC, USA). Continuous variables were expressed by median and range and tested using the Mann–Whitney U test. Categorical data were analyzed using Fisher’s exact test or the Chi-squared test as appropriate. Bonferroni corrections were applied to correct for multiple tests. *P*-values < 0.05 were considered as statistically significant.

## Results

### Clinical characteristics

The clinical characteristics of the eligible patients are presented in Table 1. No significant differences were observed in age, sex, tumor location, tumor histology, pathological stage, and frequency of dMMR between P-MA and MA groups.

**Table 1:** Clinical characteristics of the enrolled patients

	P-MA group n = 323	MA group n = 199	*P*-value
**Age at surgery, years, median (range)**	70 (39–95)	71 (32–95)	0.74
**Male/female, n**	190/133	114/85	0.80
**Family history of colorectal cancer, n (%)**			0.65*
**Yes**	121 (37.5)	71 (35.7)	
**No**	174 (53.9)	106 (53.3)	
**Unknown**	28 (8.7)	22 (11.1)	
**Tumor location, n (%)**			0.90*
**Right colon**	120 (37.2)	78 (39.2)	
**Left colon**	80 (24.8)	50 (25.1)	
**Rectum**	123 (38.1)	71 (35.7)	
**Tumor histology, n (%)**			0.98****
**Tubular adenocarcinoma**	295 (91.3)	182 (91.4)	
**Poorly differentiated adenocarcinoma**	5 (1.5)	2 (1.0)	
**Mucinous**	16 (5.0)	12 (6.0)	
**Others**	7 (2.2)	3 (1.5)	
**Pathological stage, n (%)**			0.83***
**0**	4 (1.2)	6 (3.0)	
**I**	77 (23.8)	50 (25.1)	
**II**	95 (29.4)	53 (26.6)	
**III**	114 (35.3)	71 (35.7)	
**IV**	33 (10.2)	19 (9.5)	
**Frequency of dMMR, %**	8.0	13.1	0.09

Pathological stage was defined according to the UICC-TNM, 8th edition

dMMR, deficient mismatch repair; MA, multistep approach; P-MA, pre-multistep approach

*P*-value < 0.05

* Chi-squared test with Bonferroni correction: *P*-value < 0.017

** Chi-squared test with Bonferroni correction: *P*-value < 0.013

*** Chi-squared test with Bonferroni correction: *P*-value < 0.010

The frequency of dMMR in all patients was 10.0%. The site of the primary tumor was most often the right colon. In addition, dMMR was not observed in Stage IV. The expression of both MLH1 and PMS2 was lost in 45 patients, whereas the expression of MSH2 and MSH6 was lost in 3 patients. Moreover, isolated loss of PMS2 and MSH6 expression was observed in one and two patients, respectively. Nonetheless, the loss of expression of all four MMR proteins was observed in one patient. However, the loss pattern of any MMR protein expression was not statistically different between the two groups (Table 2).

**Table 2:** Clinical characteristics of deficient mismatch repair cases

	P-MA group n = 26	MA group n = 26	*P*-value
**Age at surgery, years, n (%)**			0.37
**< 70**	6 (23.1)	10 (38.5)	
**70**	20 (76.9)	16 (61.5)	
**Sex, male/female**	12/14	16/10	0.40
**Family history of colorectal cancer, n (%)**			0.60*
**Yes**	6 (23.1)	6 (23.1)	
**No**	19 (73.1)	20 (76.9)	
**Unknown**	1 (3.8)	0 (0)	
**Synchronous colorectal cancer, n (%)**	1 (3.8)	3 (11.5)	0.61
**Metachronous colorectal cancer, n (%)**	4 (15.4)	4 (15.4)	1.00
**Tumor location, n (%)**			0.99*
**Right colon**	24 (92.3)	22 (84.6)	
**Left colon**	1 (3.8)	2 (7.7)	
**Rectum**	1 (3.8)	2 (7.7)	
**Tumor histology, n (%)**			0.91**
**Tubular adenocarcinoma**	15 (57.7)	19 (73.1)	
**Poorly differentiated adenocarcinoma**	1 (3.8)	1 (3.8)	
**Mucinous**	6 (23.1)	4 (15.4)	
**Others**	4 (15.4)	2 (7.7)	
Pathological stage*, m (%)			0.25***
**0**	1 (3.8)	1 (3.8)	
**I**	3 (11.5)	9 (34.6)	
**II**	15 (57.7)	12 (46.2)	
**III**	7 (26.9)	4 (15.4)	
**IV**	0 (0)	0 (0)	
**MMRP IHC, n**			0.97***
**MLH1 and PMS2 deficient**	23 (88.5)	22 (84.6)	
**MSH2 and MSH6 deficient**	1 (3.8)	2 (7.7)	
**PMS2 deficient only**	0 (0)	1 (3.8)	
**MSH6 deficient only**	2 (7.7)	0 (0)	
**Null phenotype**	0 (0)	1 (3.8)	

Pathological stage was defined according to the UICC-TNM, 8th edition

MMRP, mismatch repair protein; IHC, immunohistochemistry; MA, multistep approach; P-MA, pre-multistep approach

P-value < 0.05

* Chi-squared test with Bonferroni correction: P-value < 0.017

** Chi-squared test with Bonferroni correction: P-value < 0.013

*** Chi-squared test with Bonferroni correction: P-value < 0.010

### P-MA

In P-MA group, only 2 of the 26 dMMR patients received genetic counseling, and 1 of them underwent genetic testing. When the remaining 24 patients were asked why they did not receive genetic counseling, the most frequent response was “Do not wish to have a detailed examination due to advanced age” (11 cases). (Table 3).

**Table 3:** Interview results of patients who did not receive genetic counseling during the pre-multistep approach with multidisciplinary team management period

Main reason for not receiving genetic counseling	n = 24
**Do not wish to have a detailed examination due to advanced age, n (%)**	11 (45.8)
**No interest in inherited colorectal cancer, n (%)**	5 (20.8)
**No family history of Lynch syndrome-associated cancer, n (%)**	2 (8.3)
**Insufficient explanation of inherited colorectal cancer, n (%)**	2 (8.3)
**Childless, n (%)**	2 (8.3)
**No money to have genetic testing, n (%)**	1 (4.2)
**Hospital death, n (%)**	1 (4.2)

### MA

**Fig. 2:**
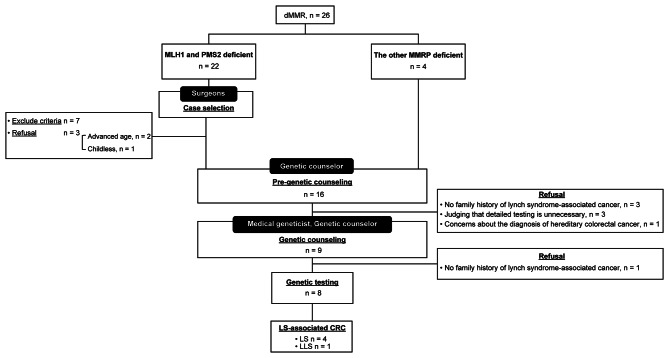
The flow chart of universal screening for Lynch syndrome in the multidisciplinary team management In the case of expression loss of both MLH1 and PMS2, after case selection by surgeons, the eligible patients received a step-by-step explanation about the genetic disease in pre-genetic and genetic counseling. In the case of a protein expression loss pattern other than MLH1 and PMS2, all patients received a step-by-step explanation about the genetic disease in pre-genetic and genetic counseling dMMR: deficient MMR, LS: Lynch syndrome, LLS: Lynch-like syndrome, CRC: colorectal cancer

Figure 2 presents the results of pre-genetic counseling, genetic counseling, and genetic testing in the MA group. The expression of both MLH1 and PMS2 was lost in 22 patients. Seven patients of the MLH1 and PMS2 deficient cases were excluded by exclusion criteria. Of the 19 eligible patients, 16 received pre-genetic counseling. At pre-genetic counseling, family history was re-investigated, and three patients with no family history of LS-associated cancer did not receive genetic counseling. The remaining four patients also decided not to undergo genetic counseling after understanding the overview of hereditary CRC. After pre-genetic counseling, nine patients received genetic counseling and eight underwent genetic testing. Finally, four patients were diagnosed with LS and one with Lynch-like syndrome (LLS).

### Assessment of MA performance

The rates of genetic counseling and genetic testing were significantly higher in MA group than in P-MA group. The diagnosis rate of LS-associated hereditary CRC was also significantly higher in MA group (P-MA group, 0.3%; MA group, 2.5%; *P* = 0.03) (Table 4).

**Table 4:** Comparison of genetic counseling, genetic testing, and the incidence of Lynch syndrome-associated colorectal cancer before and after multistep approach with multidisciplinary team

	P-MA group n = 26	MA group n = 26	*P*-value
**Pre-genetic counseling, n (%)**	NA	16 (61.5)	
**Genetic counseling, n (%)**	2 (7.7)	9 (34.6)	0.04
**Genetic test, n (%)**	1 (3.8)	8 (30.8)	0.02
**Hereditary colorectal cancer, n (%)**	1 (3.8)	5 (19.2)	0.19
**Lynch syndrome, n (%)**	1 (3.8)	4 (15.3)	0.32*
***MLH1***	1	2	
***PMS2***	0	1	
***MSH2***	0	1	
**Lynch-like syndrome**	0	1	1.00
**The incidence of Lynch syndrome-associated colorectal cancer (%)**	0.3	2.5	0.03

*P*-value < 0.05

* Chi-squared test with Bonferroni correction: *P*-value < 0.017

## Discussions

This study evaluated the changes in the rates of genetic counseling and genetic testing as well as the diagnostic rate of LS before and after MA. Comparing the two study groups in MA, the addition of surgeon-led case selection and step-by-step explanations by a genetic counselor and medical geneticists significantly increased the rate of patients receiving genetic counseling and genetic testing. As a result, LS-associated CRC diagnosis rates also increased dramatically. In addition, MA could be performed without problems despite the small number of medical and human genetics specialists. To our knowledge this is the first study to show the effect of a multidisciplinary team intervention of universal screening for LS of Japanese.

The first point is on case selection. To compensate for the small number of medical and human genetics specialists, it was necessary to focus on appropriate cases. We excluded patients with first-episode CRC aged above 70 years without family history from the dMMR cases to improve the diagnostic efficiency of LS-associated CRC. Our interview results of P-MA group indicated that “Do not wish to have a detailed examination due to advanced age” was the most common response. Boundaries based on age are often discussed for universal screening for LS. Although dMMR CRC tends to be more frequent among those with advanced age-onset CRC, LS-associated CRC tends to be rarely included among them [14, 15]. Screening patients under a certain age (e.g., aged < 70 years) has been proposed, considering the efficiency and cost-effectiveness. However, the use of a cut-off age of < 70 years for LS screening causes 15% of the LS cases to be missed [16]. Therefore, we considered age-only boundaries to be inappropriate for universal screening. The leading cause of sporadic dMMR CRC with advanced age is acquired aberrant methylation of the promoter region of the *MLH1* gene [17]. We thought that case selection was necessary for patient with the expression loss of both MLH1 and PMS2. On the contrary, CRC in patients with LS is characterized by the development of synchronous/metachronous tumors [12, 13, 18, 19]. Considering the disease characteristics of LS for the elderly, in addition to family history, the criteria were whether they had synchronous/metachronous tumors or not. With this case selection, we previously reported that it was possible to diagnose LS even in patients aged above 70 years [20]. In this way, we think that MA is a cost-effective approach in terms of picking up appropriate cases based on disease characteristics of LS.

The second point is on step-by-step explanations in pre-genetic and genetic counseling. In pre-genetic counseling, the genetic counselors primarily provide psychological care, such as explaining an outline of hereditary diseases and listening to the patient’s concerns [21]. In genetic counseling, the medical geneticist and genetic counselor explain the specialized details of the disease. The step-by-step explanations allow for acceptance regarding the genetic disease. We consider these steps very important to complement Japanese patients’ lack of knowledge about genetic diseases. According to a report from a large academic medical center, despite the 100% referral to a genetic counselor, the subsequent rates of genetic counseling and genetic testing were 71% and 66%, respectively [22]. This data highlights that patients play a role in their diagnosis through the perception of genetic disease. Our interview results of P-MA group indicated that “No interest in hereditary colorectal cancer” and “Insufficient explanation of hereditary colorectal cancer” were the top responses, except for “Do not wish to have a detailed examination due to advanced age.” Without providing correct knowledge about genetic diseases, the patients themselves may make their own judgments or feel highly anxious about genetic diseases. By providing step-by-step explanations, 89% of the patients who received genetic counseling despite the patient’s free choice understood the need for testing and underwent a genetic test in our study.

The diagnosis rate of LS in MA group was significantly higher than in P-MA group and comparable to previous reports [23, 24]. On the contrary, in Japan, the reported incidence rate of LS among CRC was 0.4–0.7%, which was lower than that of MA group of this study [25, 26]. As noted that the detection of LS in clinical practice would be limited in a recent cohort study in Japan, the percentage of genetic testing was 44.4% [26], which was lower than that in our study. It was possible that many LS cases were missed in their study.

This study had several limitations. First, and most importantly, it was a single-center and small sample investigation. Our participants represent a clinic-based population from a single geographic region, thus limiting the generalizability of our experience and findings. Second, measurement bias may have been existed because this study was a retrospective observational study. Third, although there was no statistical difference between the two groups, MA group had more dMMR cases than P-MA group. The reason for the high rate of dMMR cases was unknown in the MA group, but it might influence the diagnosis rate of LS-associated CRC. Fourth, we have not evaluated the diagnostic accuracy of LS-associated CRC by MA; it was unknown whether LS-associated CRC was present or absent among the 18 patients with MA group who did not undergo genetic testing. Moreover, there may be new barriers to LS diagnosis created by MA. In particular, future study is needed on whether the introduction of our case selection is possible to omit *BRAF* V600E mutation or *MLH1* promoter methylation analysis and whether step-by-step explanations given to complement patients’ lack of knowledge about genetic diseases may have a negative impact, such as causing further anxiety to patients. Fifth, during the subject period, the antibodies used in IHC changed, but this was due to a change in equipment at the facility. The differences in antibodies might influence the diagnosis rate of LS-associated CRC.

In conclusion, this study highlights the importance of MA after universal screening to increase the diagnosis rate of LS-associated CRC. This study contributes to the gastrointestinal genetic practice in Japan because it can be a model case for a multidisciplinary team from universal screening to the diagnosis of LS-associated CRC. We consider the feasibility study of the introduction of MA to other institutions is needed in the future.

## Electronic supplementary material

Below is the link to the electronic supplementary material.

Supplementary Material 1

## Data Availability

All data generated or analyzed during this study are included in this published article [and its supplementary information files].

## List of abbreviations

LS: Lynch syndrome
CRC: colorectal cancer
MA: Multistep approach with multidisciplinary team
IHC: immunohistochemistry
MMR: mismatch repair
MMRP: dMMR:mismatch repair proteins
dMMR: deficient mismatch repair
